# Leukocytes with chromosome Y loss have reduced abundance of the cell surface immunoprotein CD99

**DOI:** 10.1038/s41598-021-94588-5

**Published:** 2021-07-26

**Authors:** Jonas Mattisson, Marcus Danielsson, Maria Hammond, Hanna Davies, Caroline J. Gallant, Jessica Nordlund, Amanda Raine, Malin Edén, Lena Kilander, Martin Ingelsson, Jan P. Dumanski, Jonatan Halvardson, Lars A. Forsberg

**Affiliations:** 1 Department of Immunology, Genetics and Pathology and Science for Life Laboratory, Uppsala University, Uppsala, Sweden; 2 Department of Medical Sciences, Science for Life Laboratory, Uppsala University, Uppsala, Sweden; 3 Department of Public Health and Caring Sciences / Geriatrics, Uppsala University, Uppsala, Sweden; 4 Faculty of Pharmacy, 3P Medicine Laboratory, International Research Agendas Programme, Medical University of Gdańsk, Gdańsk, Poland; 5 The Beijer Laboratory, Uppsala University, Uppsala, Sweden

**Keywords:** Gene expression analysis, Immunological techniques, Proteomic analysis, RNA sequencing, Predictive markers, Diseases, Biomarkers

## Abstract

Mosaic loss of chromosome Y (LOY) in immune cells is a male-specific mutation associated with increased risk for morbidity and mortality. The *CD99* gene, positioned in the pseudoautosomal regions of chromosomes X and Y, encodes a cell surface protein essential for several key properties of leukocytes and immune system functions. Here we used CITE-seq for simultaneous quantification of *CD99* derived mRNA and cell surface CD99 protein abundance in relation to LOY in single cells. The abundance of CD99 molecules was lower on the surfaces of LOY cells compared with cells without this aneuploidy in all six types of leukocytes studied, while the abundance of CD proteins encoded by genes located on autosomal chromosomes were independent from LOY. These results connect LOY in single cells with immune related cellular properties at the protein level, providing mechanistic insight regarding disease vulnerability in men affected with mosaic chromosome Y loss in blood leukocytes.

## Introduction

Men with LOY carry a fraction of circulating immune cells without the Y chromosome and this aberration is detectable in at least 10% of the peripheral blood cells in about 10–40% of 60–80 year old men^1–5^. Moreover, a study of men over 90 years of age showed that more than half of the subjects displayed LOY in blood leukocytes^6^ and single cell analyses of blood cells from old men diagnosed with Alzheimer’s disease identified leukocytes without chromosome Y in every studied subject^7^. These results establish mosaic LOY as the most common somatic mutation in hematopoietic lineages of aging men. Furthermore, the frequency of blood cells with LOY typically increase over time within serially studied men^1,8–10^. In addition to age, germline genetic susceptibility^2–4,11^ and environmental exposures such as smoking^3,8,11,12^ are replicated risk factors for LOY in peripheral blood cells.

Men affected with LOY display an increased risk for all-cause mortality^1,12^ and conditions such as various forms of cancer^1,10,13–17^, autoimmune disease^18,19^, Alzheimer’s disease^5^, cardiovascular events^12,20^, diabetes^12^ and age-related macular degeneration^21^. The increased risk for pathology and mortality associated with LOY could be one of the reasons why men live on average about 5 years shorter lives compared to women^1,5,22,23^. Even though the list of diseases showing association with LOY has been growing continuously, the mechanism(s) by which LOY in blood might be connected with pathology in other organs remain understudied. Genome-wide association studies identified up to 156 risk variants for LOY^2–4,11^ and interestingly, an overlap in genetic susceptibility for various conditions in males and females^2^. This ‘common soil’ of genetic predisposition suggest that LOY in blood could be viewed as a barometer of general genomic instability and thus, likely reflecting risk for disease processes in somatic tissues. In parallel, a more direct link with accelerated pathology would be conceivable, if normal immune functions are negatively affected by LOY in leukocytes^1,2,5–7,22,23^. This hypothesis is supported by studies describing involvement of chromosome Y in transcriptional regulation and various functions in leukocytes^4,7,24–30^ challenging the view of the male sex chromosome as a ‘genetic wasteland’. The importance of normal expression of Y-linked genes was shown by association between extreme down-regulation of chromosome Y genes (EDY) in patients with cancer^31^ as well as Alzheimer’s disease^32^. Outside of the Y chromosome, transcriptome analyses of peripheral leukocytes identified almost 500 autosomal genes showing LOY associated transcriptional effects (LATE) including genes involved in immune functions and other biological processes, likely disturbing cellular homeostasis^7^. Moreover, men diagnosed with prostate cancer were primarily affected with LOY in T-lymphocytes and granulocytes while Alzheimer’s disease patients displayed higher levels of LOY in NK cells^7^. In aggregate, the results from recent studies suggest that LOY in the hematological system of aging men is not phenotypically neutral.

One of the immune genes showing LATE, by consistent downregulation in LOY cells, is the *CD99* gene positioned in pseudoautosomal region 1 (PAR1) of chromosomes X and Y^33^. *CD99* escapes X-inactivation in females, indicating the importance of its balanced expression^34,35^. In males, previous transcriptome analyses of single cells and bulk sorted cellular populations found that the level of CD99 mRNA transcripts were lower in all studied types of leukocytes with LOY, such as NK cells, monocytes, T- and B-lymphocytes^7^. It is likely that the reduced expression of *CD99* is directly linked with the copy number change at this locus due to the aneuploidy, while expression of the X-linked copy is retained^7^. CD99 is a transmembranous glycoprotein found at low levels in most tissues and highly expressed in cell types such as hematopoietic progenitor cells, peripheral blood cells and endothelial cells^36,37^. Its normal functions was recently reviewed^36^ and notably, when present at the cell surface, this protein is essential for the process of transendothelial migration (TEM) in which immune cells cross vascular walls through a sequence of interactions with endothelial cells^38^. Likewise, cell surface CD99 is involved in cell–cell adhesion that facilitates immune cell interactions^39,40^. Furthermore, intracellular CD99 regulates post-Golgi trafficking and transport of proteins to the plasma membrane^41^. For example, the cell surface abundance of human leukocyte antigen (HLA class I) and T-cell receptor (TCR) as well as major histocompatibility complex (MHC class I and II), have been linked with CD99 function^41,42^. Moreover, associations between LOY and blood cell counts was recently reported in human populations^4,28^. It is possible that altered cell differentiation could be connected with LOY associated dysregulation of CD99, since in vitro studies of hematopoietic progenitors suggest a role for CD99 in normal immune cell differentiation, selection and apoptosis^43–45^. Given the previously described reduced level of *CD99* mRNA in leukocytes with LOY and its vital immunological roles; we sought here to study in vivo collected leukocytes to investigate if LOY also affect the cell surface abundance of the functionally relevant CD99 protein.

## Results and discussion

**Figure 1:**
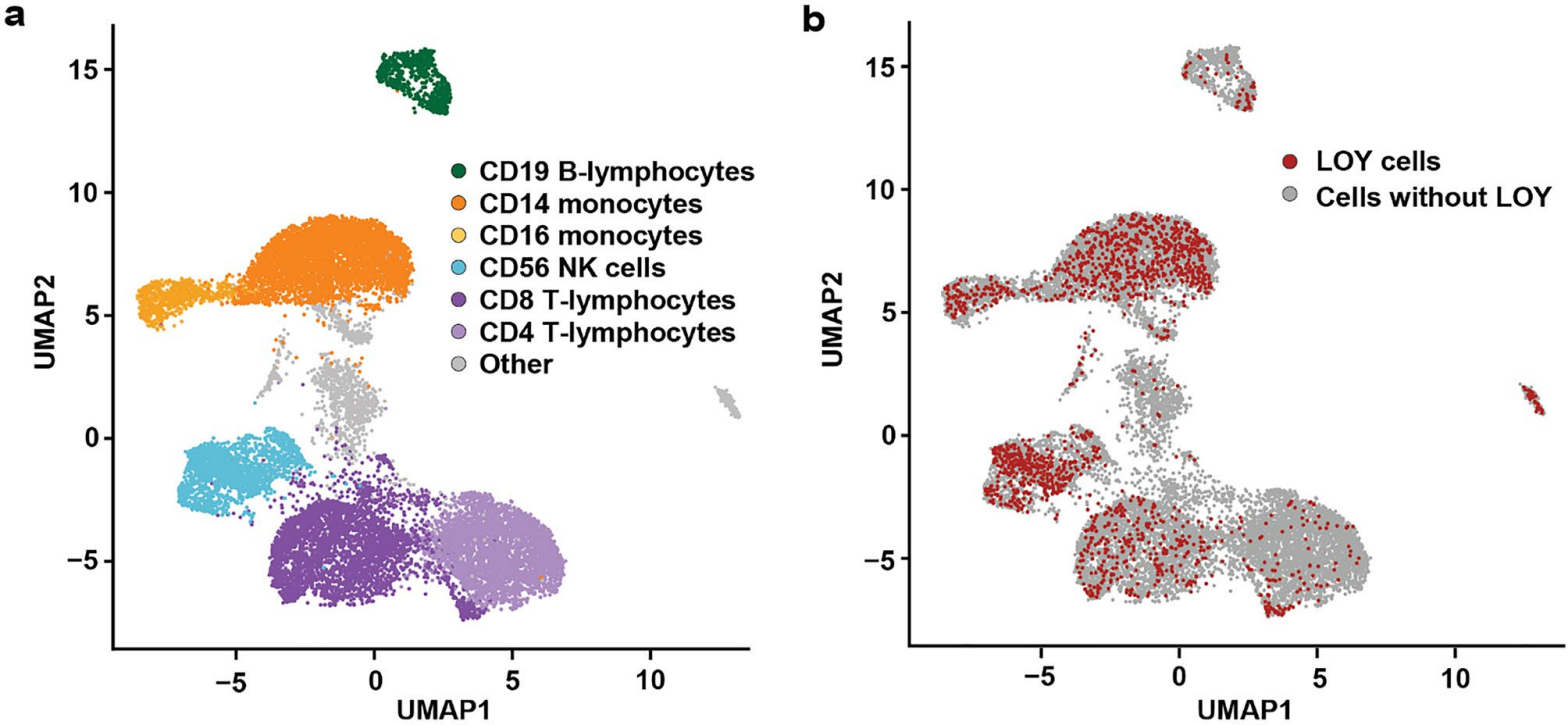
Occurrence of chromosome Y loss in different types of leukocytes visualized using UMAP based on CITE-seq single cell mRNA sequencing readout. Panel (**a**) displays six types of leukocytes labeled by color and named by the cell surface protein marker targeted in CITE-seq protocol. Grey color denotes single cells of other types of leukocytes, not targeted at the protein level by the applied assay. Panel (**b**) illustrates the distribution of single cells classified as LOY cells (red) as well as normal cells (grey). The percentage of LOY within each cell type were: 5.6% in CD19 B-lymphocytes, 15.2% in CD14 monocytes, 15.0% in CD16 monocytes, 20.9% in CD56 NK cells, 8.6% in CD8 T-lymphocytes and 2.4% in CD4 T-lymphocytes.

From freshly collected blood samples, we studied the abundance of CD99 cell surface protein as well as CD99 mRNA in single cells with and without LOY using Cellular Indexing of Transcriptomes and Epitopes by sequencing (CITE-seq)^46^. The method combines a droplet-based high-throughput single-cell RNA sequencing technology with oligonucleotide-labelled antibodies targeting cell surface proteins. By incubating the cells with the antibodies prior to droplet generation, the oligonucleotide labels are indexed with the same cell specific barcodes as the mRNA during sequencing library preparation, and can thus be quantified and traced to the cell of origin after sequencing. CITE-seq therefore provide both transcriptional and phenotypical information simultaneously at the single cell level^46^. In the CITE-seq protocol applied here, RNA expression and protein level readouts were generated for individual leukocytes by combining 10X Genomics 3’ transcriptome single cell solution v.2 with antibody-linked sequence tags for cell surface protein markers. After sequencing, a pooled dataset including 14,376 single cells originating from four male subjects diagnosed with Alzheimer’s disease (median age 81.5 years) was established. Single cell identities were determined from RNA expression profiles and visualized using Uniform Manifold Approximation and Projection (UMAP) (Figs. 1, S1). Consistent results derived from the single cell experiments encompassing the pooled dataset suggest comparability between batches and individuals (Fig. S1). In addition to the CITE-seq construct targeting Y-linked CD99 protein, we also applied antibody constructs targeting six cell surface proteins encoded by autosomal genes; i.e. CD19, CD14, CD16, CD56, CD8 and CD4. These markers are normally present on the cell surfaces of B-lymphocytes, classic and non-classic monocytes, NK cells, CD8 + T-lymphocytes and CD4 + T-lymphocytes, respectively. The occurrence of these cell surface proteins on the studied leukocytes is visualized Fig. S2 and confirm the mRNA-based cell type identification and clustering.

**Figure 2:**
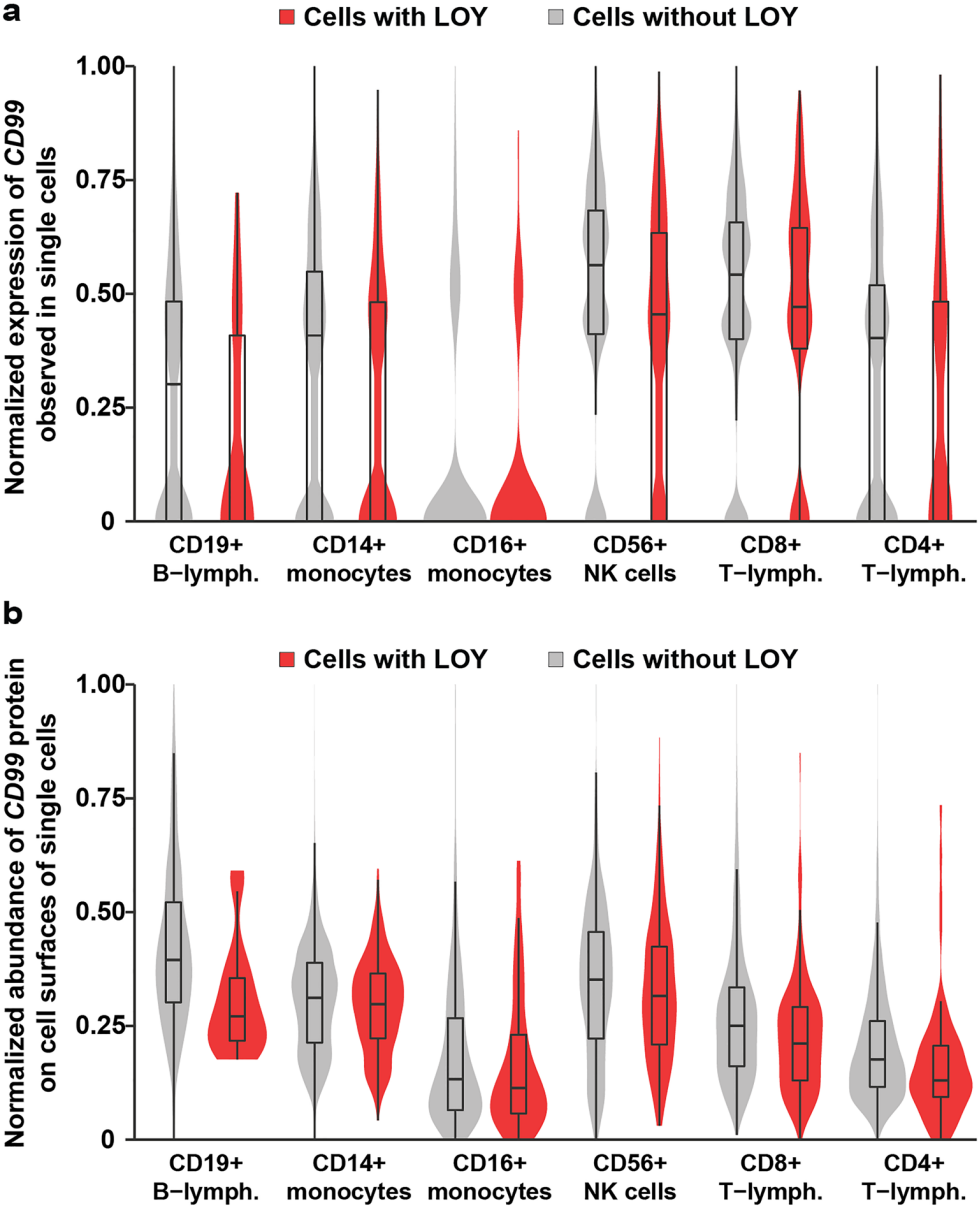
CITE-seq results illustrating a reduced abundance of CD99 analytes in single cells with LOY. The measurements of mRNA and protein abundances displayed on the Y-axes in panels (**a**) and (**b**) were linearly adjusted on a scale between 0 and 1 before plotting, to increase interpretation and comparability between cell types. Overlapping violin- and box-plots were used to illustrate the distributions of CD99 derived mRNA (panel **a**) as well as cell surface CD99 protein (panel **b**) in cells classified as LOY cells (red) and normal control cells with an intact Y chromosome (grey).

**Figure 3:**
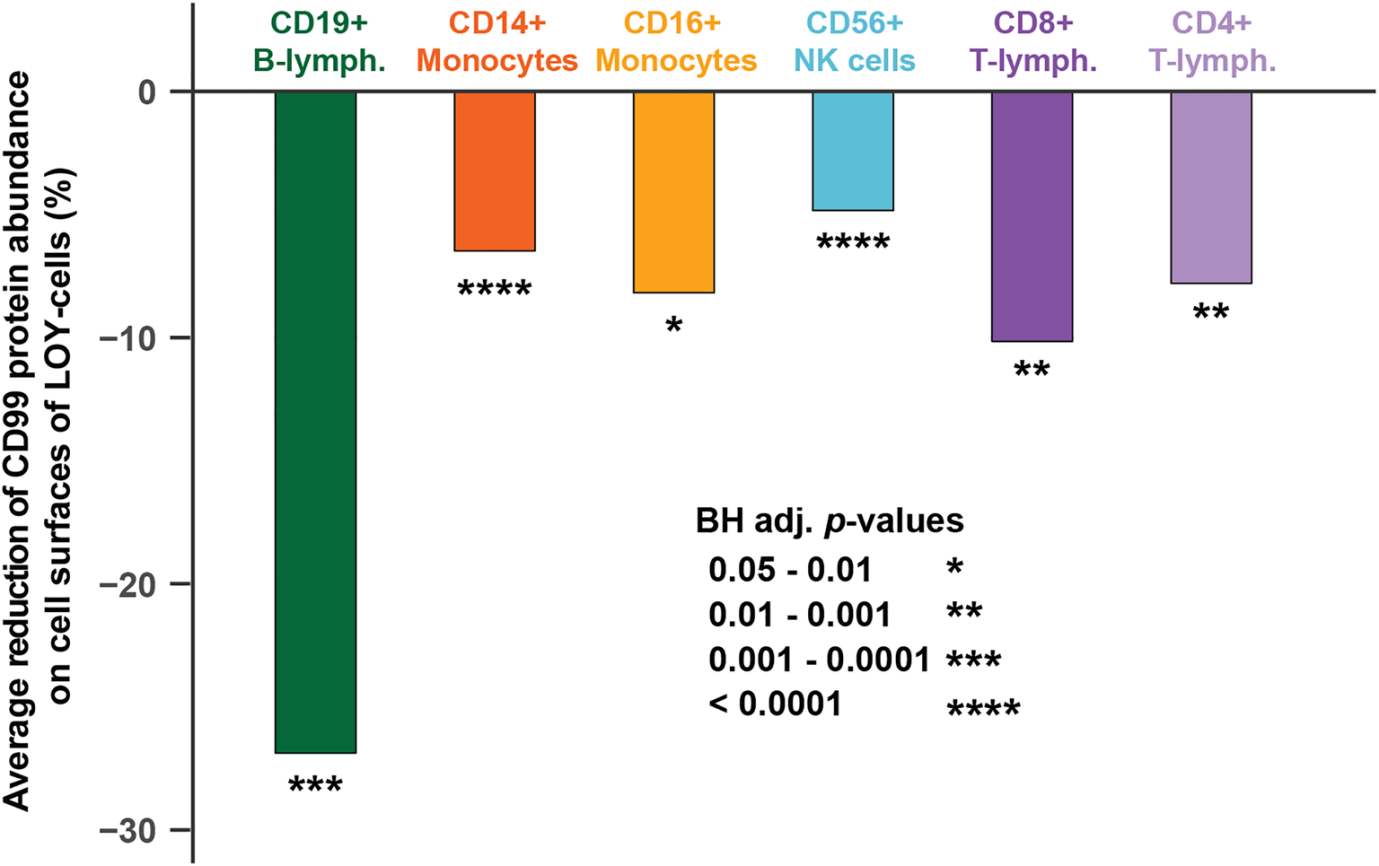
Reduced abundance of CD99 protein on the surface of LOY cells by cell type. The Y-axis display the average decrease in protein level observed in single cells with LOY compared with normal cells and expressed as percentage change. The percentage of change was calculated by transformation of the average log fold change estimated by *Seurat*, to improve interpretation. Asterisks denote adjusted significance levels after Benjamini-Hochberg (BH) correction for multiple testing.

Next, the LOY status of each sequenced cell was determined from the transcriptome data by the lack of expression of genes located in the male-specific region of chromosome Y (MSY), as described previously^2,7^. Occurrence of single cells with LOY was observed in all subjects and in all types of studied leukocytes, ranging from 2.4 to 20.9% in frequency between different cell types (Figs. 1, S1, Table S1). To test if the abundances of *CD99* derived mRNA and CD99 protein display alterations in cells with LOY, compared with normal cells without the aneuploidy, we first performed logistic regression using the pooled dataset in models adjusted for confounders such as cell donor, experimental batch, UMI counts, percentage of mitochondrial RNA and cell type. These primary analyses of CD99 abundance showed a significant overall reduction in the level of mRNA (*Z* = -7.6, *p* = 3.66e-14) as well as cell surface protein levels (*Z* = -12.6, *p* < 2e-16) in single cells with LOY. Further exploratory analyses showed that the reduction of CD99 protein was present in all types of studied leukocytes with LOY (Figs. 2, 3, Table 1). The largest reduction was observed in B-lymphocytes with an average log fold change between single cells of -0.31 (adj. *p* = 0.0006) representing a 27% decrease of CD99 protein abundance on the surface of B-lymphocytes with LOY. In contrast to the significant reduction of CD99 protein levels, the abundance of the six cell surface CD proteins encoded by autosomal genes investigated were not affected by LOY in any of the cell types studied (Fig. S3, Table 1).

**Table 1:** Reduced abundance of immunoprotein CD99 in leukocytes lacking chromosome Y. Quantification of mRNA transcripts and seven cell surface proteins (CD99, CD19, CD14, CD16, CD56, CD8 and CD4) in single cells using CITE-seq. LOY associated changes was identified by comparing the abundance of transcripts and proteins in LOY cells compared with normal cells.

Analyte and cell type	Changes in mRNA levels in single cells with LOY			Changes in protein levels in single cells with LOY		
	log FC	%	Adj. *p*	log FC	%	Adj. *p*
CD99 in CD19 + B-lymphocytes	–0.32	–27.6	0.9876	–0.31	–26.8	0.0006***
CD99 in CD14 + Monocytes	–0.23	–20.2	0.0002***	–0.07	–6.5	< 0.0001****
CD99 in CD16 + Monocytes	–0.12	–11.2	0.9394	–0.09	–8.2	0.0316*
CD99 in CD56 + NK cells	–0.17	–15.7	0.0041**	–0.05	–4.8	< 0.0001***
CD99 in CD8 + T-lymphocytes	–0.05	–4.7	0.3384	–0.11	–10.2	0.0062**
CD99 in CD4 + T-lymphocytes	–0.09	–8.7	1	–0.08	–7.8	0.0015**
CD19 in CD19 + B-lymphocytes	–0.08	–7.7	0.9587	–0.20	–18.3	0.8538
CD14 in CD14 + Monocytes	0.06	5.8	1	0.04	3.6	0.9187
CD16 in CD16 + Monocytes	–0.01	–0.5	0.3142	0.17	18.9	0.3633
CD56 in CD56 + NK cells	0.004	0.4	0.9982	0.08	8.2	1
CD8 in CD8 + T-lymphocytes	–0.04	–3.4	0.7613	–0.13	–12.1	0.3348
CD4 in CD4 + T-lymphocytes	–0.07	–6.3	1	0.002	0.2	1

"log FC" is the observed log fold change in abundance and "%" show the LOY associated changes as percentages. The "Adj. *p*" refers to Benjamini–Hochberg corrected *p*-values in statistical models adjusted for relevant confounders and asterisks denote level of significance.

The RNA-readout also displayed an overall reduction in expression of CD99 mRNA in single cells with LOY and exploratory analyses showed that the downregulation was significant in CD14 + monocytes and CD56 + NK cells (Table 1). This result validates previous results showing a reduced *CD99* expression in LOY cells^7^. However, compared with the general decrease of CD99 protein abundance on cell surfaces, we observed a greater variation in the CD99 mRNA abundance between single cells (Fig. 2). For example, a substantial proportion of single cells in our assay displayed no expression of CD99 mRNA transcripts, highlighting the issue of zero-inflation commonly observed in single cell mRNAseq data. In contrast, the cell surface abundance of the CD99 protein displayed a more even distribution and a significant reduction in single cells with LOY. This result supports the view that proteins constitute more stable markers for cellular properties of single cells, compared with the generally more fluctuating mRNA levels^46–48^.

From a translational perspective, the possible functional consequences of LOY and CD99 deficiency in leukocytes are promising. Previous studies have shown that CD99 and PECAM-1 are independently responsible for interactions necessary for TEM, specifically the passage of leukocytes through endothelial junctions^38^. In tests where monoclonal antibodies were used to block cell surface CD99 in monocytes; TEM was severely inhibited in vitro^38^ as well as in vivo^49^, with monocytes arrested partway through the junction. Other functional studies show an impact on cell-to-cell adhesion of lymphocytes after blocking CD99 with monoclonal antibodies^39,40^. Furthermore, CD99 regulates transport of proteins to the plasma membrane. For example, low abundance of CD99 in B-lymphocytes was associated with reduced cell surface levels of MHC class I proteins; a deficiency that could be restored by increasing CD99 abundance^41^. Moreover, blocking CD99 resulted in the intracellular accumulation of MHC class I molecules in B- as well as T-lymphocytes^41,42^. On the other hand, engagement of CD99 increased the abundance of the immunoproteins TCR and MHC class I and II molecules on the surface of human T-lymphocyte progenitors^50^, further supporting the importance of CD99 for physiological intracellular protein transport. CD99 has also been shown to be involved in regulation of apoptosis and differentiation of developing B- and T-lymphocytes^44,45,51^. These studies show that cell death could be induced by the ligation of monoclonal antibodies to CD99. Another study further show that the cell surface level of CD99 affected the developmental trajectories of human hematopoietic progenitors^43^. For example, B-lymphocytes were mainly produced by hematopoietic progenitors with high CD99 levels. Interestingly, recent studies suggest an association between LOY and blood cell counts in human populations^4,28^ that might be connected with dysregulation of CD99 in progenitors with LOY. In aggregate, the results from functional studies, together with our results showing an overall reduction of CD99 connected with LOY, suggest that this aneuploidy could have direct impact on leukocyte physiology.

In summary, men carrying circulating immune cells without the Y chromosome display an increased risk for disease and mortality. Here we demonstrate that single cells with LOY show a reduced abundance of CD99 protein, encoded by a gene located on chromosomes X and Y. This cell surface immunoprotein is a key molecule for leukocyte properties such as transendothelial migration, adhesion, differentiation, apoptosis as well as intracellular trafficking of proteins involved in immune surveillance. These results provide proof-of-concept for the detection of a disease associated protein on single cells with LOY and support the hypothesis that LOY in leukocytes could be directly connected with impaired immune functions, via disruption of physiological CD99 biology. This result, however, do not exclude other potential LOY-related disease mechanisms. Nonetheless, a direct role of LOY in immune cells on increased disease vulnerability in affected men would benefit the establishment of LOY as a predictive biomarker; a possibility that require further research and additional functional validation.

## Methods

### Generation of CITE-seq probes

The antibodies used for detection of different target proteins (Table S2) were buffer exchanged to PBS using 7 MWCO Zeba columns (ThermoFisher, USA) and concentrated to 1 µg/µl using Amicon 30 kDa spin columns (Merck). The antibodies were conjugated to azide modified DNA oligonucleotides (Table S2) using DBCO-NHS ester cross-linker (Sigma-Aldrich) using cross-linker:antibody ratio 30:1 and oligonucleotide:antibody ratio 3.33:1. After confirming successful conjugation with polyacrylamide gel electrophoresis, NaN3 (0.04% final concentration) was added to conjugates to quench further conjugation. Antibody-DNA conjugates were pooled at equal ratios. Unconjugated oligonucleotides were removed using Amicon 100 kDa spin columns (Merck).

### Samples and cell preparations

Whole blood was collected from four aging men from an Alzheimer’s disease cohort at the Geriatric/Memory Clinic, Uppsala Academic Hospital in Sweden. The median age of the subjects was 81.5 years (range 70–83). The donors were included based on sample availability and had not previously been studied with regard to LOY status. PBMCs were isolated from whole blood samples using BD Vacutainer CPT tubes (BD Biosciences, USA) according to the manufacturer’s instruction. The isolated PBMCs were quantified and assessed for viability using an EVE cell counter (NanoEnTek, Seoul) and diluted in PBS with 0.04% UltraPure™ BSA (Thermo Scientific) for a final concentration of 10 million cells per millilitre. One million cells per sample were treated with Fc Receptor blocking solution (Human TruStain FcX, BioLegend) and incubated with the pooled antibody-conjugates (1 µg of each antibody) as previously described^46^. The study was performed in accordance with relevant guidelines and regulations and was approved by the local research ethics committee in Uppsala, Sweden (Regionala Etikprövningsnämnden i Uppsala (EPN), Dnr: 2013/350) and informed consent was obtained from all participants.

### Library preparation and sequencing

Sequencing libraries were prepared using Chromium Single Cell 3’ v2 protocol CG00052 (10X Genomics) with modifications described in the detailed protocol CITE-seq_190213 (cite-seq.com/protocol)^46^. Two libraries (from subjects UAD100 and UAD101) were first sequenced in a pilot batch and then re-sequenced together with two additional libraries (from subjects UAD104 and UAD105) in a second batch. The RNA and protein libraries were pooled 19:1 and sequenced on a HiSeq2500 instrument in the pilot study and using NovaSeq S1 flow cell (Illumina, USA) in the second batch, according to manufacturer’s instructions.

### Bioinformatic analyses

After sequencing, the raw base calls for each sample were de-multiplexed and mapped to the hg19 version of the human genome or the oligonucleotide sequences linked with specific antibody tags using Cellranger v2.0.2 (10X Genomics)^52^. Following this, RNA reads mapping to the human genome were quantified using Cellranger. Reads mapping to the antibody derived tags of each investigated protein were counted using CITE-seq-Count, with the recommended settings for 10X-derived sequencing libraries (https://github.com/Hoohm/CITE-seq-Count). We used R (version 3.6.1) applying the package *Seurat* (version 3.0.0)^53,54^ and the data from all samples were pooled into a single Seurat object for further analyses. The package *Future* (version 1.12.0, https://github.com/HenrikBengtsson/future) was used to multi-thread *Seurat* functions. Quality assessment was performed for each single cell and was based on three following criteria; number of expressed genes, number of unique molecular identifiers (UMI) and percentage of mitochondrial reads. Specifically, to reduce the risk of including droplets containing more than one cell per droplet, all observations of more than 2000 expressed genes were excluded. To avoid inclusion of dead or low quality cells, at least 2500 UMI counts were required. Furthermore, the percentage of reads originating from mitochondrial genome was quantified and only cells showing 1.5–5% mitochondrial content were considered normal. The three cut-offs used in quality check were defined from visualization of data using histograms. For the 14,376 cells passing quality control, we performed normalizations for the RNA and protein assays separately in *Seurat*. For the RNA, the function *Log normalized* was used to a scale factor of 10,000, while for the protein assay data we applied the function *Centered log-ratio*.

### Clustering and identification of cell types

The clustering was performed on the RNA-assay using the 1000 most variable features (i.e. expressed genes) based on the Seurat function *FindVariableFeatures*. To minimize variation due to technical factors, the included features were scaled based on number of UMI, percentage of mitochondrial RNA, library prep-batch and sequencing batch. Principle component analysis was performed to cluster based on only the most explanatory features. Hence, 36 principle components evaluated using functions *JackStrawPlot* and *ElbowPlot* and of these, the first 22 were subsequently used for clustering. We implemented *FindNeighbors* method and *FindClusters* with 0.6 resolution to identify clusters and visualized the results using Uniform Manifold Approximation and Projection applied in the package *UMAP* (version 0.3.8, https://github.com/tkonopka/umap). To determine the type of leukocytes within each of the 14 clusters predicted we applied an in-house script using expression of previously known cell type specific markers. Next, the six cell types targeted by the CITE-seq constructs was identified by plotting the protein-derived data upon the RNA-based clusters using the *Viridis* package (version 0.5.1, https://sjmgarnier.github.io/viridis) and validated using heatmaps.

### Determination of LOY in single cells

LOY status in each single cell was determined as described previously^2,7^. Briefly, all genes located on the Y chromosome were retrieved from Ensembl (v.99)^55^ using the *BioMart* package (v.2.40.0)^56^. The sum of all features in the RNA-assay, with HGNC-symbols matching those on the Y chromosome, was calculated for each cell. Each sequenced cell with expression of autosomal genes, but without transcripts from genes located in the male-specific region of chromosome Y (MSY) was considered as LOY cells.

### Analyses of phenotypical effects in LOY cells

The Seurat wrapper *FindMarkers* was used to estimate the average log fold change in RNA and protein abundances between LOY and normal cells. This was done separately for each cell type. The parameters in *FindMarkers* for minimum fold-change threshold and fraction of cells with any expression was set zero in order to capture the maximal amount of information from all features in the models. A summary of relevant single cell metrics including the number of cells, reads and percentage of LOY per subject is provided in Table S3.

### Statistical analyses

For the *FindMarkers* wrapper, the MAST algorithm (version 1.9.2) was used for the RNA assay, which is specifically developed to handle the zero-inflation aspect of scRNAseq data. Logistic regression was the model implemented for the protein assay, as it did not suffer from zero-inflation. Both *FindMarker* models were corrected for batch effects (library preparation and sequencing run), number of UMI and percentage mitochondrial reads. All p-values, from each cell type and assay test, were adjusted together using Benjamini–Hochberg correction for multiple testing. Logistic regression models implemented by the *glm* function in R was used for tests of overall CD99 abundance in relation to LOY in singe cells. The same covariates as used in the *FindMarkers* tests described above, with the binary outcome described by the model set as LOY-status.

## Supplementary Information

Supplementary Information.

## Data Availability

The data that support the findings of this study are available from the corresponding author upon reasonable request.
