# Supplementary material for: Leukocytes with chromosome Y loss have reduced abundance of the cell surface immunoprotein CD99
Source: Sci Rep. 2021 Jul 26;11:15160. doi: 10.1038/s41598-021-94588-5 (PMC8313698; doi:10.1038/s41598-021-94588-5)
Supplement: Supplementary file 1 — Supplementary Information. [file 41598_2021_94588_MOESM1_ESM.pdf]

Supplementary Information:

Leukocytes with chromosome Y loss have reduced abundance of the cell surface immunoprotein CD99

Mattisson et al.

Contents: Page:

Contents:	Page:
Fig. S1	2
Fig. S2	3
Fig. S3	4
Table S1	5
Table S2	6
Table S3	6

Figure S1

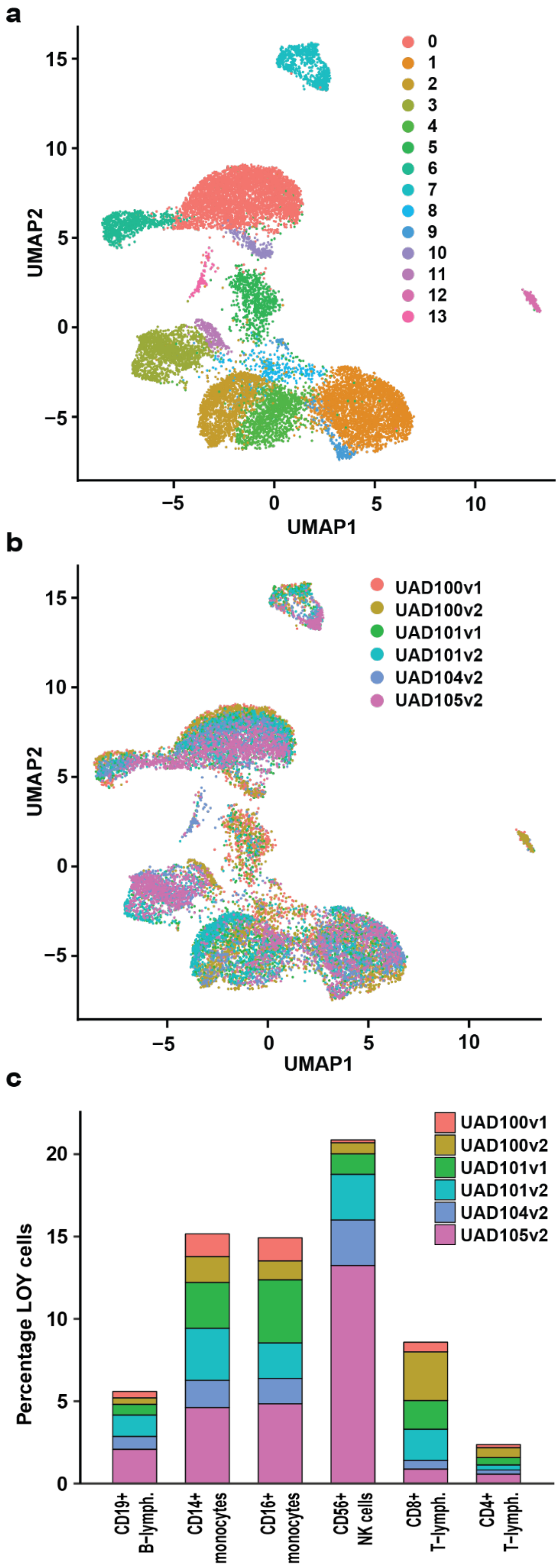

Supplementary figure 1. Clustering, batch effects and LOY fractions; aspects of the data used for description and analyses. Panel **a** display a UMAP where cells have been coloured by the clustering performed by *Seurat* based on gene expression profiles of single cells. Cell type prediction was performed separately for each of these 14 clusters using expression profiles of known cell type markers. From these predictions, the cell types targeted by the CITE-seq constructs was identified. Panel **b** display the same UMAP as in panel **a**, with cells coloured based on the donor of blood samples. The first part of the sample names are identifier for the sampled patient and the second part is batch related, i.e. v1 or v2 in the end of the name indicates in which sequencing run the sample was sequenced. Panel **c** display the percentage of LOY cells per cell type using bar plot. Each bar is split based on the fraction of LOY cell in different cell types within each sample, marked by the same colour as in the previous panel.

Figure S2

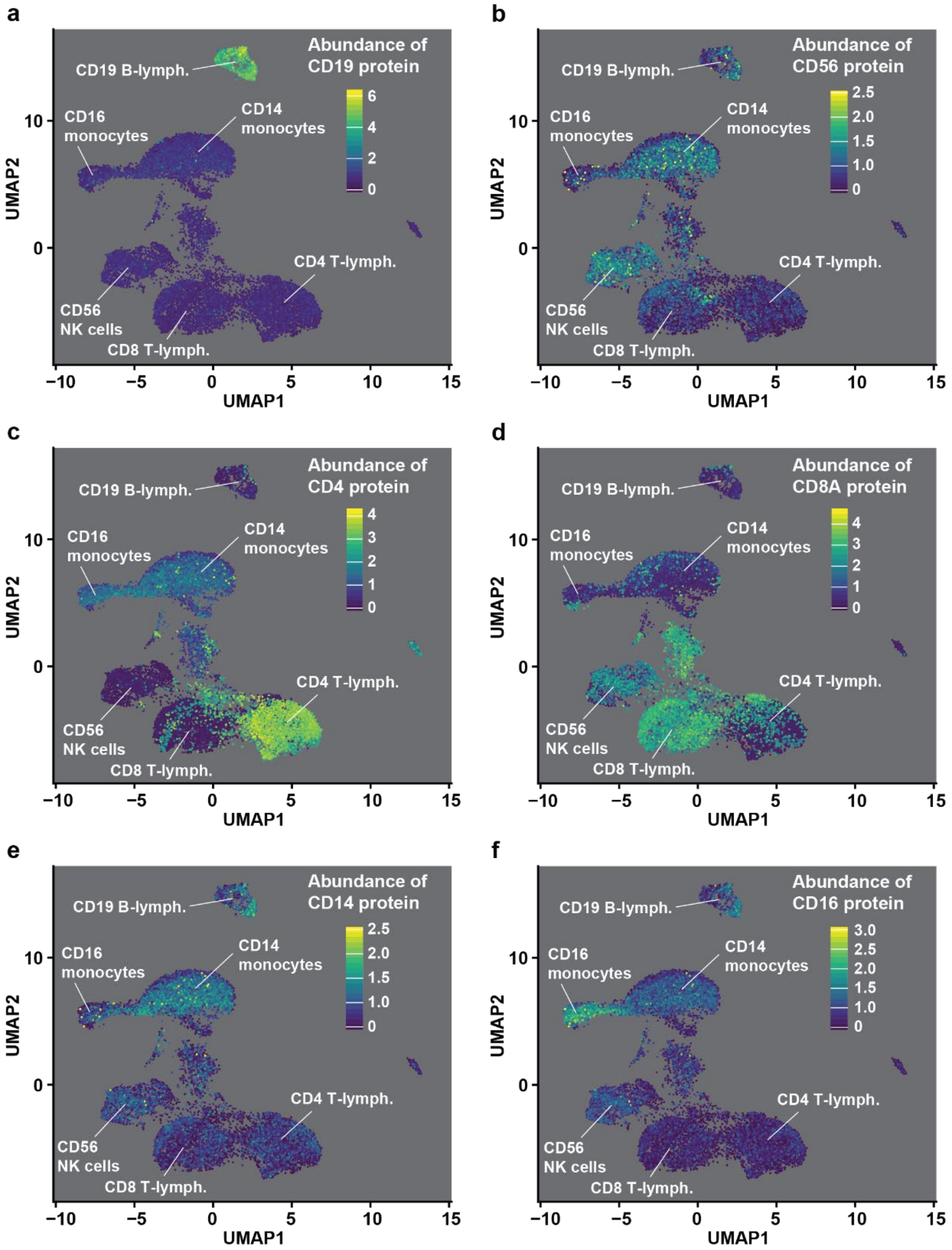

Supplementary figure 2. Concordance between the RNA-based cell type classification and corresponding cell surface proteins targeted by the applied CITE-seq protocol. Each panel display a UMAP in which single cells have been coloured based on the abundance of cell surface protein markers characteristic for the studied cell types, i.e. the levels of CD19, CD56, CD4, CD8, CD14 and CD16 are displayed in panels **a-f**, respectively. Higher protein abundance translates into brighter colour, used to validate the cell type predictions by the RNA assay.

Fig. S3

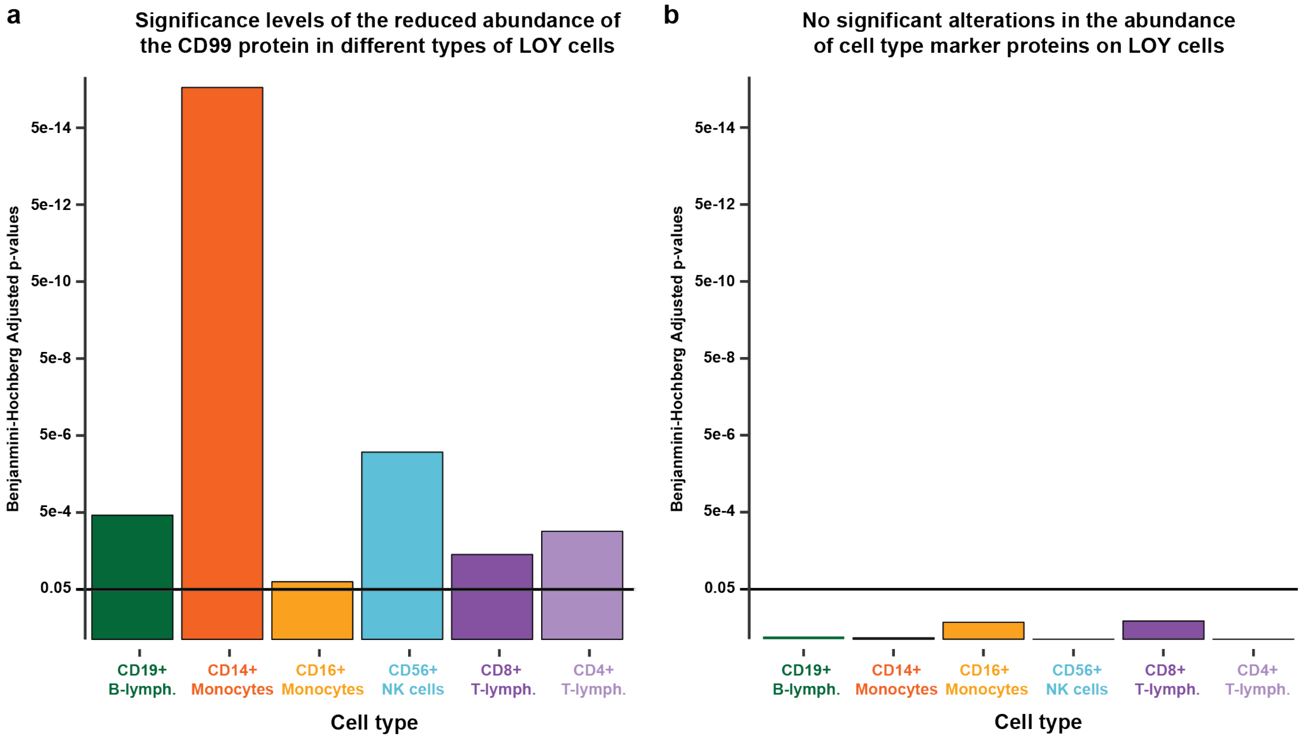

Supplementary figure 3. Results from analyses of cell surface abundance of seven CD proteins in relation to LOY in single cells. On the Y-axes are plotted the adjusted p-values from *FindMarker* tests comparing the average amount of cell surface protein on single cells with LOY to cells with a Y chromosome. Panel **a** illustrates a significant reduction of CD99 molecules in all studied cell types. Panels **b** display the corresponding non-significant results from the other cell surface protein measured, i.e. CD19, CD56, CD4, CD8, CD14 and CD16.

Table S1. The number and fraction of single cells with LOY in six types of leukocytes measured using CITE-seq.

**Cell type**	**Number of LOY cells**	**Number of normal cells**	**Fraction of cells with LOY (%)**
CD19+ B-lymphocytes	43	726	5.6
CD14+ Monocytes	627	3508	15.2
CD16+ Monocytes	117	667	15.0
CD56+ NK cells	369	1399	20.9
CD8+ T-lymphocytes	322	3430	8.6
CD4+ T-lymphocytes	75	3093	2.4

Table S2. Details of antibodies and the oligonucleotides used for producing the CITE-seq probes. The nucleotide “B” is a mixed base code for G, C or T and the symbol “*” is a code for phosphorothioates.

Antibody specificity	Antibody Vendor	Catalog number	Clone	Oligo Vendor	Oligo ID	Sequence
CD16	BioLegend	360702	B73.1	IDT	CSv3_ADT1	/5AzideN/CCTTGGCACCCGAGAATTCCACATGATTGGCTCBAAAAAAAAAAAAAAAAAAAAAAAAAAAA*A*A
CD56	BioLegend	304602	MEM-188	IDT	CSv3_ADT2	/5AzideN/CCTTGGCACCCGAGAATTCCAGAGGCGATTGATBAAAAAAAAAAAAAAAAAAAAAAAAAAAA*A*A
CD4	BioLegend	344602	SK3	IDT	CSv3_ADT3	/5AzideN/CCTTGGCACCCGAGAATTCCATGTCCGGCAATABAAAAAAAAAAAAAAAAAAAAAAAAAAAA*A*A
CD19	BioLegend	302202	HIB19	IDT	CSv3_ADT4	/5AzideN/CCTTGGCACCCGAGAATTCCATGGTGAACCTGGBAAAAAAAAAAAAAAAAAAAAAAAAAAAA*A*A
CD8A	BioLegend	344702	SK1	IDT	CSv3_ADT5	/5AzideN/CCTTGGCACCCGAGAATTCCAGATCGTAATACCBAAAAAAAAAAAAAAAAAAAAAAAAAAAA*A*A
CD14	BioLegend	301802	M5E2	IDT	CSv3_ADT6	/5AzideN/CCTTGGCACCCGAGAATTCCAAAGCGCTTGGCABAAAAAAAAAAAAAAAAAAAAAAAAAAAA*A*A
CD99	BioLegend	318002	HCD99	IDT	CSv3_ADT8	/5AzideN/CCTTGGCACCCGAGAATTCCAGTCTAGACTTCGBAAAAAAAAAAAAAAAAAAAAAAAAAAAA*A*A

Table S3. Metrics describing the final, QC filtered, single-cell dataset per sample. Each sample is identified by subject library and sequencing batch. Values are presented as the total amount, a median of cells or a percentage of cells.

Sample	Number of reads from mRNA in CITE-seq	Number of reads from protein in CITE-seq	Total number of studied cells	Median number of genes per cell (mRNA)	Median UMI count per cell (mRNA)	Median UMI count per cell (protein)	Number of B lymphocytes	Number of CD14+ Monocytes	Number of CD16+ Monocytes	Number of NK cells	Number of CD8+ T lymphocytes	Number of CD4+ T lymphocytes	Percentage of studied cells with LOY (%)
UAD100v1	5758906	1278994	1423	1180	3837	766	115	447	138	55	382	286	7.2
UAD100v2	11538950	2296744	2981	1154	3619	763	133	642	115	175	1101	815	7.3
UAD101v1	9186599	3101692	2433	1203	3566	971	131	808	150	151	712	481	10.3
UAD101v2	11661596	3779339	2987	1297	3655	887	145	934	127	315	913	553	9.6
UAD104v2	7753403	2810261	1968	1187	3724	887,5	84	514	124	376	317	553	8.3
UAD105v2	10393582	3464955	2584	1348	3778	670,5	161	790	130	696	327	480	20.5
